# Can calculation of energy expenditure based on CO_2_ measurements replace indirect calorimetry?

**DOI:** 10.1186/s13054-016-1595-8

**Published:** 2017-01-21

**Authors:** Taku Oshima, Séverine Graf, Claudia-Paula Heidegger, Laurence Genton, Jérôme Pugin, Claude Pichard

**Affiliations:** 1 Clinical Nutrition, Geneva University Hospital, Rue Gabrielle-Perret-Gentil 4, 1211 Geneva 14, Switzerland; 2 Adult Intensive Care, Geneva Universtiy Hospital, Rue Gabrielle-Perret-Gentil 4, Geneva 14, 1211 Switzerland

**Keywords:** Indirect calorimetry, Energy expenditure, Accuracy, Critical care

## Abstract

**Background:**

Methods to calculate energy expenditure (EE) based on CO_2_ measurements (EEVCO_2_) have been proposed as a surrogate to indirect calorimetry. This study aimed at evaluating whether EEVCO_2_ could be considered as an alternative to EE measured by indirect calorimetry.

**Methods:**

Indirect calorimetry measurements conducted for clinical purposes on 278 mechanically ventilated ICU patients were retrospectively analyzed. EEVCO_2_ was calculated by a converted Weir’s equation using CO_2_ consumption (VCO_2_) measured by indirect calorimetry and assumed respiratory quotients (RQ): 0.85 (EEVCO_2__0.85) and food quotient (FQ; EEVCO_2__FQ). Mean calculated EEVCO_2_ and measured EE were compared by paired *t* test. Accuracy of EEVCO_2_ was evaluated according to the clinically relevant standard of 5% accuracy rate to the measured EE, and the more general standard of 10% accuracy rate. The effects of the timing of measurement (before or after the 7th ICU day) and energy provision rates (<90 or ≥90% of EE) on 5% accuracy rates were also analyzed (chi-square tests).

**Results:**

Mean biases for EEVCO_2__0.85 and EEVCO_2__FQ were -21 and -48 kcal/d (*p* = 0.04 and 0.00, respectively), and 10% accuracy rates were 77.7 and 77.3%, respectively. However, 5% accuracy rates were 46.0 and 46.4%, respectively. Accuracy rates were not affected by the timing of the measurement, or the energy provision rates at the time of measurements.

**Conclusions:**

Calculated EE based on CO_2_ measurement was not sufficiently accurate to consider the results as an alternative to measured EE by indirect calorimetry. Therefore, EE measured by indirect calorimetry remains as the gold standard to guide nutrition therapy.

## Background

Indirect calorimetry is the gold standard method to determine energy expenditure (EE) and guide nutrition therapy in critically ill patients in order to avoid deleterious under- or overfeeding [1–3]. Indirect calorimeters analyze respiratory gases of the patients to measure their oxygen consumption (VO_2_) and carbon dioxide production (VCO_2_) and derive EE by the Weir’s equation [4]. The ratio of VCO_2_ to VO_2_ (VCO_2_/VO_2_), called the respiratory quotient (RQ), is considered as an indicator of measurement adequacy (i.e. <0.67 and >1.3 require careful interpretation) [5] and of substrates oxidation in stable state subjects [6]. However, indirect calorimetry is not conducted routinely in most intensive care units (ICU) mainly due to the lack of a reliable indirect calorimeter, and manpower with appropriate expertise to conduct and analyze the results [7]. Recent studies comparing currently available calorimeters have demonstrated that the measured EE is variable from one calorimeter to another [8, 9], although they are still more consistent than the EE calculated from predictive equations [10].

Methods to calculate EE from CO_2_ measurements (EEVCO_2_) derived from mechanical ventilators have been proposed as a surrogate to indirect calorimetry [11, 12]. This simplified method measures only the VCO_2_ derived from measurements of exhaled gas volume and CO_2_ concentrations. The approach assumes that the RQ value is either a fixed value (e.g. 0.85) [12] or equal to the food quotient (FQ) [11], i.e. the estimated RQ resulting from the oxidation of energy substrates. Recent studies have demonstrated that EEVCO_2_ estimates EE of critically ill patients more accurately than predictive equations [11, 12]. However, the validity of this method as an alternative to indirect calorimetry is questionable, since the variability of RQ demonstrated in previous literature is likely to influence the accuracy of the EEVCO_2_ calculation [5].

This study aims at determining whether EEVCO_2_ can be considered an alternative to EE measured by indirect calorimetry in critically ill patients on mechanical ventilation.

## Methods

### Study design

This retrospective observational study includes the measurements of indirect calorimetry performed in the mixed medical-surgical adult ICU at the Geneva University Hospital, Switzerland.

### Data collection

We included 278 mechanically ventilated ICU patients from the institutional database of indirect calorimetry, conducted by the Clinical Nutrition Department. We collected data regarding physical characteristics (age, gender, height, anamnestic weight, body mass index), diagnosis and severity at ICU admission (primary diagnosis, Acute Physiology and Chronic Health Evaluation (APACHE) II score [13], Simplified Acute Physiology Score (SAPS) II [14]), and patient observations at the time of the measurement (body temperature, Glasgow coma scale, Sedation-Agitation Scale [15]). We also reported the characteristics of energy provision at the time of the indirect calorimetry, consisting of the route of administration (enteral nutrition (EN), parenteral nutrition (PN), combined (EN + PN), or non-nutritional energy sources), the total administered energy (kcal/d) along with the composition of the energy substrates (carbohydrate, protein, lipids). Energy from glucose-containing solutions and propofol was included in the calculation of the total administered energy.

### Indirect calorimetry

All measurements were conducted using the Deltatrac Metabolic Monitor® (Datex, Helsinki, Finland) which was calibrated before each measurement according to the manufacturer’s procedure. Calorimetry was not conducted in patients with ventilator settings exceeding the general limits (fraction of inhaled O_2_ (FiO_2_) < 60%, positive end expiratory pressure (PEEP) < 9cmH_2_O), or with contraindicated treatments (e.g. chest drains for pneumothorax, inhaled nitric oxide). Patients were in resting state for at least 1 hour prior to the measurement, i.e. free of physical activity, mobilization, and transport. Measurements were conducted to obtain valid recordings for at least 20 minutes in a single measurement session for each patient after reaching steady state; recordings from the first 5 minutes of measurements and from non-stable states were excluded. As the Deltatrac® was a stand-alone device, minute-by-minute readings of VO_2_ (ml/min STPD) and VCO_2_ (ml/min STPD) were obtained as printouts or handwritten recordings. The results were reported in the clinical database as the means of valid recordings for the duration of the measurement. Mean VO_2_ and mean VCO_2_ of each patient was used for the individual calculation the RQ (=VCO_2_/VO_2_) and the determination of the measured EE by the modified Weir’s equation:

$$ \mathrm{E}\mathrm{E}\ \left(\mathrm{kcal}/\mathrm{d}\right) = 1.44 \times \left[3.941 \times {\mathrm{VO}}_2\left(\mathrm{mL}/ \min \right) + 1.11 \times {\mathrm{VCO}}_2\left(\mathrm{mL}/ \min \right)\right] $$

Fraction of inspired O_2_ (FiO_2_, %), and minute volume of ventilation (L/min BTPS) at the time of the indirect calorimetry were also collected. The day of the measurement was recorded as the number of days after ICU admission.

### EEVCO_2_ calculation

The EEVCO_2_ was calculated using VCO_2_ values measured by indirect calorimetry, and two different RQ values: fixed value of 0.85 (EEVCO_2__0.85) and FQ (EEVCO_2__FQ).

#### Calculation of EEVCO_2__0.85

By assuming an RQ = 0.85 [12], the Weir’s equation can be rewritten as:

$$ {\mathrm{EEVCO}}_2\_0.85\left(\mathrm{kcal}/\mathrm{d}\right)=1.44\times \left[3.941\times {\mathrm{VCO}}_2\left(\mathrm{mL}/ \min \right)/0.85+1.11\times {\mathrm{VCO}}_2\left(\mathrm{mL}/ \min \right)\right] $$

#### Calculation of EEVCO_2__FQ

The FQ calculation was based on the O_2_ utilization and CO_2_ production during energy substrate oxidation [5]. Carbohydrates are oxidized for energy as:

C_6_H_12_O_6_+ 6O_2_ → 6CO_2_+ 6H_2_O + 679 kcal.

The ratio of produced CO_2_ to consumed O_2_, or FQ, is therefore equal to 1.0 (6 CO_2_/6 O_2_ = 1).

Fat oxidation is described by the following the reaction (e.g. palmitic acid):

CH_3_(CH_2_)14COOH + 23O_2_ → 16CO_2_+ 16H_2_O + 2,395 kcal

The results depend on the type of fatty acid, but on average the FQ will be 0.7 [5]. Protein oxidation is not as simple to express in a formula, as they are not always completely oxidized in the body. The FQ for protein is 0.8 [5]. By assuming that RQ is equal to FQ, the Weir’s equation can be rewritten as following:

$$ {\mathrm{EEVCO}}_2\_\mathrm{F}\mathrm{Q}\left(\mathrm{kcal}/\mathrm{d}\right)=1.44\times \left[3.941\times {\mathrm{VCO}}_2\left(\mathrm{mL}/ \min \right)/\mathrm{F}\mathrm{Q}+1.11\times {\mathrm{VCO}}_2\left(\mathrm{mL}/ \min \right)\right] $$

VCO_2_ was obtained from indirect calorimetry measurement, and FQ was calculated from the composition of the energy sources actually administered at the time of the measurement, including energy sources related to drug administration [11].

### Data analysis and statistics

Patient characteristics are presented as mean ± standard deviation (SD), median (interquartile range), or number (percentage) as appropriate. Normality of distribution was confirmed based on the skewness and kurtosis analyses prior to parametric analyses. We used paired *t* tests to compare calculated EEVCO_2_ and EE measured by indirect calorimetry, and chi-squared tests for comparisons of proportions. The agreement between the measured EE and EEVCO_2_ were analyzed using Bland-Altman plots. The individual accuracy of EEVCO_2_ in comparison to measured EE was expressed as 5% accuracy rates defined as the proportion of calculated EEVCO_2_ values within the clinically relevant limits, i.e. 5% of the measured EE. The results were also evaluated by 10% accuracy rates, a more general standard of used in previous literature [11].

EEVCO_2_ accuracy rates were further tested according to the timing of measurement, i.e. within or after the 7th ICU day, and the rate of energy provision, i.e. <90 or ≥90% of measured EE. These factors were selected to test the hypothesis that patients treated for longer duration and fed closer to the energy targets are metabolically more stable and make better candidates for EEVCO_2_ [11].

All statistical analyzes were conducted on IBM SPSS Statistics® software version 22 (IBM, Corp., Armonk, NY, USA). Statistical significance was defined as *p* values <0.05.

## Results

### Patient characteristics

Patients’ characteristics are shown in (Table 1). Patients with a wide variety of primary diagnoses were analyzed, reflecting patient recruitment in a polyvalent ICU. Ninety-nine percent of the patients were fed with nutrition formulas at the time of the measurement, mainly through the enteral route (90%, including EN + PN). The remaining patients received energy only from non-nutrition energy source, i.e. drug-related glucose or lipid administration.

**Table 1:** Patient characteristics

Patients	*n* (%)	278	(100.0)
Male		191	(68.7)
Female		87	(31.3)
Age, years		56	(18)
Height, cm		172	(9)
Anamnestic body weight, kg		76	(18)
Body mass index, kg/m^2^		25.9	(5.9)
Admission diagnosis	*n* (%)		
Shock		60	(21.6)
Multiple trauma		52	(18.7)
Neurologic		31	(11.2)
Respiratory failure		22	(7.9)
Cardiac surgery		20	(7.2)
Pneumonia		18	(6.5)
Acute pancreatitis		11	(4.0)
Myocardial infarction		10	(3.6)
Post-cardiac arrest		8	(2.9)
Liver failure		8	(2.9)
Others		38	(13.7)
APACHE II score		24	(7)
SAPS II		51	(17)
Length of ICU stay, days	median (IQR)	21	(12–37)
ICU day of evaluation, days	median (IQR)	8	(4–18)
Glasgow Coma Scale	median (IQR)	10	(8–14)
Sedation-Agitation Scale	median (IQR)	4	(3–4)
Body temperature, °C		37.3	(0.6)
FiO_2_, %		30	(7)
Minute volume, L/min BTPS		12	(3)
VO_2_, mL/min STPD		279	(61)
VCO_2_, mL/min STPD		234	(53)
Respiratory quotient		0.84	(0.09)
Energy expenditure, kcal/day		1956	(426)
Energy provision, kcal/day		1658	(696)
Energy provision rate, %		86	(35)
Nutrition route	*n* (%)		
Enteral nutrition (EN)		196	(70.5)
Parenteral nutrition (PN)		24	(8.6)
EN + PN		55	(19.8)
Non-nutrition energy sources		3	(1.1)
Food quotient		0.87	(0.01)

Results shown as mean (standard deviation) unless otherwise indicated

*APACHE* Acute Physiology and Chronic Health Evaluation, *SAPS* Simplified Acute Physiology Score, *FiO*_*2*_ fraction of inspired O_2_, *BTPS* body temperature (37 °C) ambient pressure water saturated condition, *STPD* standard temperature (0 °C) standard pressure (760 mmHg) dry condition, *VO*_*2*_ volume of O_2_ consumption, *VCO*_*2*_ volume of CO_2_ production, *IQR* interquartile range

### Accuracy of EEVCO_2_ vs. measured EE

**Fig 1:**
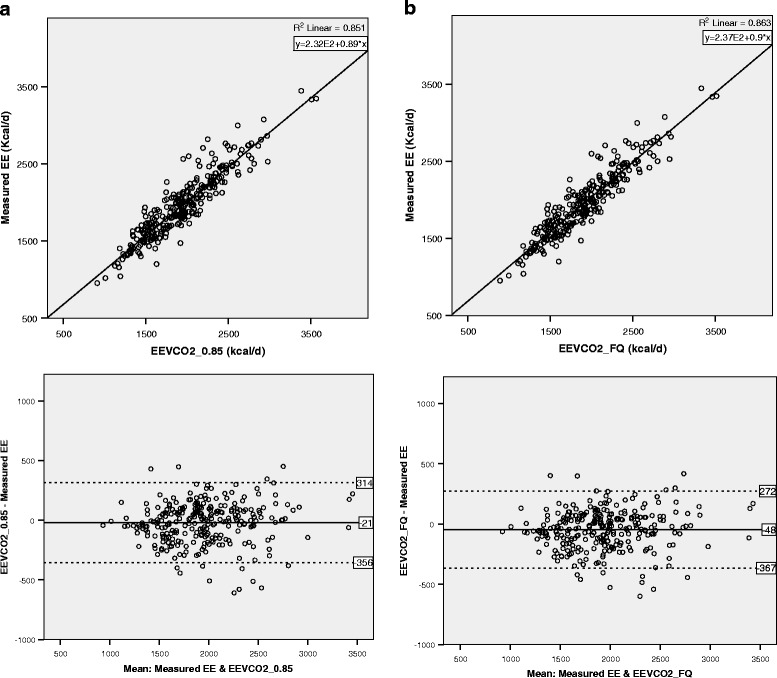
Correlation and limits of agreement between calculated energy expenditure based on CO_2_ measurements (EEVCO_2_) and energy expenditure (EE) measured by indirect calorimetry. **a** EEVCO_2__0.85 vs. measured EE. **b** EEVCO_2__FQ vs. measured EE. *Solid lines* indicate the bias and *fine dotted lines* indicate the limits of agreement in the Bland-Altman plots. *EEVCO*_2_ *_0.85* EEVCO_2_ calculated from CO_2_ consumption (VCO_2_) and assumed respiratory quotient (RQ) of 0.85, *EEVCO*_2_ *_FQ* EEVCO_2_ calculated from VCO_2_ and assuming RQ as food quotient (FQ)

Mean biases (lower, upper 95% confidence intervals) for EEVCO_2__0.85 and EEVCO_2__FQ were -21 kcal/d (-41, 1) and -48 kcal/d (-67, -28), respectively (Table 2). The limits of agreement in Bland-Altman plots were (+314, -356) and (+272, -367), respectively (Fig. 1). The 5% accuracy rates compared to measured EE were 46.0 and 46.4%, while 10% accuracy rates were 77.7 and 77.3%, respectively (Table 2).

**Table 2:** Comparison of energy expenditure based on CO_2_ measurements (EEVCO_2_) to energy expenditure (EE) measured by indirect calorimetry in mean bias and accuracy rates

	Mean, SD	Mean bias	95% CI	SD of bias	*p* value	Accuracy rates (%)	
	(kcal/d)	(kcal/d)	Lower, upper			5%	10%
EEVCO_2__0.85	1936, 441	-21	-41, 1	171	0.042	46.0	77.7
EEVCO_2__FQ	1908, 439	-48	-67, -28	163	0.000	46.4	77.3

Mean bias mean difference between EEVCO_2_ and measured EE, *p* values: paired Student *t* tests, accuracy rates proportion of EEVCO_2_ values within 5% or 10% of the measured EE

*SD* standard deviation, *CI* confidence intervals, *EEVCO*_*2*_ *_0.85* EE calculated using measured CO_2_ consumption (VCO_2_) and fixed respiratory quotient (RQ) of 0.85, *EEVCO*_*2*_ *_FQ* EE calculated using VCO_2_ and the food quotient as the RQ

#### Accuracy rates according to the timing of evaluation

There were 131 (47%) patients who were evaluated within 7 days after ICU admission, and 147 (53%) patients after the 7th day of ICU stay. There were no significant differences in the 5% accuracy rates of EEVCO_2_ based on the fixed RQ or the FQ when comparing the measurements performed before or after the 7th ICU day (Table 3).

**Table 3:** Accuracy of energy expenditure based on CO_2_ measurements (EEVCO_2_) versus energy expenditure (EE) measured by indirect calorimetry according to the timing of evaluation and energy provision rate at the time of evaluation

		*n*	EEVCO_2__0.85			EEVCO_2__FQ		
			±5% EE	(%)	*p* value	±5% EE	(%)	*p* value
Timing of evaluation	≤7 ICU day	131	58	44	0.577	59	45	0.667
	>7 ICU day	147	70	48		70	48	
Energy provision	<90% EE	139	65	47	0.810	63	45	0.718
	≥90% EE	139	63	45		66	47	

Timing of evaluation: patient stratification by measurements before or after the 7th day after ICU admission, Energy provision: patient stratification according to the proportion of energy provision at the time of measurement < 90 or ≥ 90% of EE, ±5%.

*EEVCO*_2_ *_0.85* EE calculated using measured CO_2_ consumption (VCO_2_) and fixed respiratory quotient (RQ) of 0.85, *EEVCO*_2_ *_FQ* EE calculated using measured VCO_2_ and the food quotient (FQ) as the RQ, *EE* number of patients with EEVCO_2_ values within ±5% of EE, *P* values: chi-square tests

#### Accuracy rates according to the energy provision rates at the time of evaluation

There were 139 (50%) patients who were fed < 90% of the measured EE at the time of the evaluation, and 139 (50%) patients were fed ≥ 90% of EE. There were no significant differences in the 5% accuracy rates of EEVCO_2_ based on the fixed RQ or the FQ in patients fed < 90% of the measured EE compared to those fed ≥ 90% of EE (Table 3).

## Discussion

The aim of this study was to evaluate whether EEVCO_2_ could be a considered as an alternative to measured EE in mechanically ventilated patients. Although the mean EEVCO_2_ were not considerably different from mean measured EE, 5% accuracy rates were only 46%, regardless of the RQ used for the calculation. The accuracy rates did not change for evaluations before or after the 7th ICU day, or for patients who were fed less or more than 90% of their measured EE. Therefore, we concluded that EEVCO_2_ should not be considered as an alternative to EE measured by indirect calorimetry, regardless of the time from ICU admission or the feeding status at the time of the evaluation.

### The effect of RQ on EEVCO_2_

The overall accuracy of the EEVCO_2_ calculation method is dependent on the accuracy of the assumed RQ and the VCO_2_ measurements. In the present analysis, VCO_2_ was measured by the indirect calorimeter, thus leaving RQ as the only determinant of the difference between calculated EEVCO_2_ and measured EE. The RQ can be affected by metabolic consequences related to feeding and stress response secondary to critical illness, as well as non-nutrition factors such as acid-base status. In mechanically ventilated subjects, suboptimal ventilation could lead to unstable CO_2_ elimination, resulting in erroneous representation of the metabolic status by VCO_2_. In such cases, RQ may be more correctly described as respiratory exchange ratio, as RQ refers to the gas exchange ratio of the true metabolic consequence of energy oxidation. Our study demonstrates the practical implication of the RQ variability by investigating its effect on the accuracy of EEVCO_2_ calculation.

The measured RQ was indeed variable among patients as observed in the standard deviation (±0.09) of the measured RQ (Table 1). While optimizing the conditions for VCO_2_ measurements may decrease the variability of the RQ [11], this level of variability leads to a difference of > 8% of the calculated EE. By fixing the RQ at a certain value, this variability directly results in the level of inaccuracy of the calculated EE when compared with the measured EE. The use of FQ as suggested by Stapel et al. [11] did not help to improve the EEVCO_2_ in our analysis. The method is based on the hypothesis that FQ will more accurately predict RQ, which is not often the case in critically ill patients with variable metabolic and feeding status. The standard deviation of FQ was ±0.01, demonstrating the limited possibility of agreement with the measured RQ, which presented a much larger variation. Detailed investigation by McClave et al. concluded that RQ is neither a reliable indicator of the feeding status nor strongly associated with non-nutritional factors such as condition of ventilation and acid-base disturbance [5], suggesting the difficulty of predicting the RQ. Thus, measuring VO_2_ by indirect calorimetry remains as the only valid solution to determine the RQ accurately.

Another important factor is that VCO_2_ has less impact on the EE compared to VO_2_. This phenomenon can be observed in the multiples for VO_2_ and VCO_2_ in the Weir’s equation (3.941 and 1.11, respectively), giving 3.6 times more weight to the VO_2_ value. As a result, VO_2_ has a much larger influence on the EE. However, the complexity of O_2_ measurements in variable high O_2_ concentration ranges precludes the VCO_2_ analyses to be accurately conducted with ventilator-derived measurements. Indirect calorimeters are specially designed to solve this issue, by installing precision O_2_ analyzers and implementing appropriate calibration procedures.

### Inaccuracy of EEVCO_2_

Mean EEVCO_2_ were in better limits of agreement to measured EE than previously reported comparisons to EE calculated by predictive equations [16]. However, individualized analysis revealed the inaccuracy hidden behind the comparison of the means. Previous studies have evaluated the accuracy of the EEVCO_2_ method according to 10% accuracy rates to measured EE [11, 12]. In fact, the 10% accuracy rates of EEVCO_2_ in our patients (77–78%) were better than in the previous study by Stapel et al. (61%) [11, 12], perhaps due to the use of VCO_2_ values measured by indirect calorimetry. In the present study, we implemented 5% accuracy rates according to the clinical relevancy of the results. For example, 5% accuracy for the mean measured EE of the present study (1956 kcal/d) means allowing ±98 kcal/d difference in the calculated EE; while it will be ±196 kcal/d for 10% accuracy. The results can vary within the ranges of 1858–2054 kcal/d for 5% accuracy, and 1760–2152 kcal/d for 10% accuracy. It is irrelevant to consider a method that allows almost 400 kcal/d difference in the calculated results as an alternative to measuring EE by indirect calorimetry. In addition, EEVCO_2_ was calculated based on the VCO_2_ measured by the indirect calorimeter, meaning that the difference in the results could only arise from the calculation based on assumed RQ. For these reasons, we decided to evaluate the validity of calculated EEVCO_2_ as an alternative to measured EE according to 5% accuracy rates.

### Timing of the measurement

We anticipated that the accuracy rates for EEVCO_2_ would be different when measured before or after the 7th day of ICU admission. This was not the case, and suggests that the metabolic state of critically ill patients is difficult to predict, even after the 7th day of ICU admission when clinicians expect that the initial stress of critical illness starts to resolve [17]. The shift between the acute and subacute (or later) phase of critical illness is generally characterized by the shift from the catabolic to anabolic condition, and complicates the metabolic pathways [18]. Prolonged immobilization and organ support therapies can also alter the metabolism, not to mention the effect of repeated stress due to secondary infections and organs failure [17, 19, 20]. Our data thus suggest that a similar variability and complicated metabolic pathways exists also during the acute phase of critical illness [21].

### The effect of energy provision

The accuracy rates of the calculated EEVCO_2_ also remained unchanged when the patients were fed less or more than 90% of their measured EE. This observation can partly be explained by the relationship between feeding and RQ, as the accuracy of EEVCO_2_ relies on the accuracy of the assumed RQ to the measured RQ. Higher rate of feeding correlates with higher RQ [5], suggesting that RQ is likely to deviate higher from the generally accepted value (i.e. 0.85) with higher rate of energy provision. At the same time, RQ is highly variable and unpredictable in critically ill patients, limiting its validity as an indicator of energy substrate oxidation [5]. Thus, variable energy provision rates can lead to variable differences between the assumed and measured RQ, leading to the inaccuracy of EEVCO_2_ calculations. The inaccuracy of EEVCO_2_ can result in misleading energy provision targets, enhancing the risk of underfeeding and overfeeding, which are both associated with a worsening of the outcome [22–24].

### Strengths and weaknesses

A major strength of the study is the large number of patients studied. Our analysis is based on 278 mechanically ventilated patients with various diagnoses, enhancing the generalizability of the results to most ICU patients. However, it should be noted that their length of stay was longer than the mean (<4 days) at our ICU. The selection of mechanically ventilated patients also means that patients had at least one organ failure [17], while the assumption of RQ in the calculation of EEVCO_2_ may be better applicable once the patients are stabilized [11].

As this was a retrospective study, the timing of the indirect calorimetry measurements was not controlled. Stratifying patients before and after the 7th ICU day may not have reflected the characteristics of acute and late-phase metabolism of ICU patients. However, we believe that this limitation can also be seen as an advantage as indirect calorimetry is usually recommended when considerable changes are observed or suspected in the patients’ conditions. In this regards, the non-significance of the stratification according to the timing of the measurement (i.e. before or after 7th ICU day) strengthened the clinical relevance of our analysis.

Another limitation arising from the retrospective nature of the study is that the analyzed measurements were conducted for clinical purpose, and not originally intended for research. Clinical conditions during the measurements such as sedation levels, ventilator types and settings, or duration of the measurements were not predefined. These factors could have affected the variability represented by the SD of the RQ, which was higher than in a previous report [11]. The VO_2_ and VCO_2_ values obtained from the clinical database were already the averaged results of the minute-by-minute readings by the Deltatrac® during the measurements, thus precluding the assessment of the stability of each measurement. We also had no comparison of 24-hour measurements, recently proposed as one of the benefits of the EEVCO_2_ method. However, it should be noted that our team members are trained to routinely conduct indirect calorimetry strictly according to our protocol, to ensure the quality of the clinical measurements.

The measurements of VCO_2_ in the present study were from indirect calorimeter, and not the measurements by mechanical ventilators as proposed in recent literature [11, 12]. We also did not conduct simultaneous measurements with EEVCO_2_ devices and indirect calorimeters. Our Deltatrac® has been used over the years, but has been regularly maintained by the Biomedical Department and has been calibrated before each measurement to assure optimal performance. The accuracy of VCO_2_ measurements of the Deltatrac® has been shown to be within 2–4% of expected values in in vitro validations [25], while the level of accuracy for VCO_2_ measurements in ventilators can vary as much as ±9%, according to the instructions manuals. Therefore, it is unlikely that the use of VCO_2_ measured by mechanical ventilators will significantly improve the accuracy of EEVCO_2_ and thus influence the conclusions of our study.

## Conclusions

Calculated energy expenditure based on CO_2_ measurements (EEVCO_2_) was not sufficiently accurate to consider the results as an alternative to measured EE by indirect calorimetry according to this retrospective study in critically ill patients. Therefore, EE measured by indirect calorimetry remains as the gold standard to guide nutrition therapy. Prospective studies are warranted to further quantify the limitations of the EEVCO_2_ method.

## Abbreviations

EE: energy expenditure
EEVCO_2_: EE calculated from VCO_2_ and assumed RQ
FQ: food quotient
RQ: respiratory quotient
VCO_2_: volume of carbon dioxide production
VO_2_: volume of oxygen consumption
