# Erratum to: Can calculation of energy expenditure based on CO2 measurements replace indirect calorimetry?

**DOI:** 10.1186/s13054-017-1650-0

**Published:** 2017-04-12

**Authors:** Taku Oshima, Séverine Graf, Claudia-Paula Heidegger, Laurence Genton, Jérôme Pugin, Claude Pichard

**Affiliations:** 1 Clinical Nutrition, Geneva University Hospital, Rue Gabrielle-Perret-Gentil 4, 1211 Geneva 14, Switzerland; 2 Adult Intensive Care, Geneva Universtiy Hospital, Rue Gabrielle-Perret-Gentil 4, Geneva14, 1211 Switzerland

## Erratum

During production of article [1], Funding and Competing Interests were published incorrectly due to a publisher error. The errors are detailed below.

## Funding

The funding section was included in the original article as below.

The study was funded by the European Society for Intensive Care Medicine (ESICM), the European Society for Clinical Nutrition and Metabolism European (ESPEN), and the public Foundation Nutrition 2000Plus, Switzerland.

This should have been as below instead.

The study was funded by the European Society of Intensive Care Medicine (ESICM), the European Society for Clinical Nutrition and Metabolism European (ESPEN), and the public Foundation Nutrition 2000Plus, Switzerland. The funding bodies were not involved in the study design, the interpretation of the results, or the drafting of the manuscript.

## Competing interests

The Competing interests section was included in the original article as below.

TO received financial support as an unrestricted academic research grant from public institutions (Geneva University Hospital) and the Foundation Nutrition 2000 Plus. CP received financial support as research grants and an unrestricted academic research grant, as well as an unrestricted research grant and consulting fees, from Abbott, Baxter, B. Braun, Cosmed, Fresenius-Kabi, Nestle Medical Nutrition, Novartis, Nutricia-Numico, Pfizer, and Solvay, outside the submitted work. The other authors declare that they have no competing interests related to the current work.

This should have been seen as below.

TO received financial support as an unrestricted academic research grant from public institutions (Geneva University Hospital) and the Foundation Nutrition 2000 Plus. CP received financial support as research grants and unrestricted academic research grants, as well as consulting fees from Abbott, Baxter, B. Braun, Cosmed, Fresenius-Kabi, Nestle Medical Nutrition, Novartis, Nutricia-Numico, Pfizer, and Solvay, outside the submitted work. TO, SG, CH, and CP are currently developing a new indirect calorimeter (The ICALIC study, ClinicalTrials.gov ID: NCT02796430). The study is supported by academic societies (see below) and the manufacturer involved (Cosmed, Italy) had no influence on the study conduct and the interpretation of the data in the current work. The other authors declare that they have no conflict of interest related to the current work.
